# Acquired genetic alterations in tumor cells dictate the development of high-risk neuroblastoma and clinical outcomes

**DOI:** 10.1186/s12885-015-1463-y

**Published:** 2015-07-10

**Authors:** Faizan H. Khan, Vijayabaskar Pandian, Satishkumar Ramraj, Mohan Natarajan, Sheeja Aravindan, Terence S. Herman, Natarajan Aravindan

**Affiliations:** 1 Department of Radiation Oncology, University of Oklahoma Health Sciences Science Center, 940 Stanton L. Young Blvd., BMSB 737, Oklahoma City, OK 73104 USA; 2 Department of Pathology, University of Texas Health Sciences Center, San Antonio, TX USA; 3 Stephenson Cancer Center, Oklahoma City, OK USA

**Keywords:** High-risk aggressive neuroblastoma, Genetic rearrangements, Karyotyping, Array CGH, Tumor progression, Clinical outcomes

## Abstract

**Background:**

Determining the driving factors and molecular flow-through that define the switch from favorable to aggressive high-risk disease is critical to the betterment of neuroblastoma cure.

**Methods:**

In this study, we examined the cytogenetic and tumorigenic physiognomies of distinct population of metastatic site- derived aggressive cells (MSDACs) from high-risk tumors, and showed the influence of acquired genetic rearrangements on poor patient outcomes.

**Results:**

Karyotyping in SH-SY5Y and MSDACs revealed trisomy of 1q, with additional non-random chromosomal rearrangements on 1q32, 8p23, 9q34, 15q24, 22q13 (additions), and 7q32 (deletion). Array CGH analysis of individual clones of MSDACs revealed genetic alterations in chromosomes 1, 7, 8, and 22, corresponding to a gain in the copy numbers of *LOC100288142, CD1C, CFHR3, FOXP2, MDFIC, RALYL, CSMD3, SAMD12-AS1,* and *MAL2*, and a loss in *ADAM5, LOC400927, APOBEC3B, RPL3, MGAT3, SLC25A17, EP300, L3MBTL2, SERHL, POLDIP3, A4GALT*, and *TTLL1.* QPCR analysis and immunoblotting showed a definite association between DNA-copy number changes and matching transcriptional/translational expression in clones of MSDACs. Further, MSDACs exert a stem-like phenotype. Under serum-free conditions, MSDACs demonstrated profound tumorosphere formation *ex vivo.* Moreover, MSDACs exhibited high tumorigenic capacity *in vivo* and prompted aggressive metastatic disease. Tissue microarray analysis coupled with automated IHC revealed significant association of RALYL to the tumor grade in a cohort of 25 neuroblastoma patients. Clinical outcome association analysis showed a strong correlation between the expression of *CFHR3*, *CSMD3, MDFIC, FOXP2, RALYL, POLDIP3, SLC25A17, SERHL, MGAT3, TTLL1*, or *LOC400927* and overall and relapse-free survival in patients with neuroblastoma.

**Conclusion:**

Together, these data highlight the ongoing acquired genetic rearrangements in undifferentiated tumor-forming neural crest cells, and suggest that these alterations could switch favorable neuroblastoma to high-risk aggressive disease, promoting poor clinical outcomes.

**Electronic supplementary material:**

The online version of this article (doi:10.1186/s12885-015-1463-y) contains supplementary material, which is available to authorized users.

## Background

Neuroblastoma (NB) is the most common cancer of infancy [1]. It originates from the sympathoadrenal lineage of the neural crest and accounts for 9.1 % of cancer-related deaths in children [2]. The clinical hallmark of NB heterogeneity is its marked variability in prognosis, ranging from spontaneous regression to an aggressive clinical course followed by death [3]. Despite intensive multimodal therapy, which may include chemotherapy, surgery, radiotherapy, myeloablative chemotherapy with autologous stem cell transplant, and/or differentiation therapy, high-risk aggressive NB remains one of the most difficult cancers to cure [4, 5]. Given its heterogeneity, resistance, and poor hematological reserve, the rate of 5 year overall survival (OS) is low (<10 %) in patients with high-risk disease, compared with 65 % 5 year OS in patients with low- or intermediate-risk disease. The rate of long-term survival is even more dismal in 10 years after diagnosis, with only 2 % OS for patients with stage 4 compared with 38–71 % for patients with low-risk disease [6, 7]. High-risk aggressive disease is typically characterized by a wide range of genomic alterations, including point mutations, copy number changes, and genetic rearrangement [8, 9]. In this study, we attempted to characterize the genetic alterations in highly malignant aggressive cells. These alterations could define the switch from favorable NB to high-risk aggressive disease.

NB is characterized by non-random chromosomal abnormalities with diagnostic and prognostic significance, including large-scale chromosomal imbalances [10–14]. Traditional cytogenetic analysis of SH-SY5Y cells successfully described some abnormalities [15–17]. Array comparative genomic hybridization provided additional molecular cytogenetic insights into the SH-SY5Y cell line karyotype [18, 19]. Comprehensive molecular cytogenetic approaches that reveal the presence of previously undetected allelic imbalances and copy number variations are usually well-suited to studying segmental rearrangements, such as the deletion of 1p or 11q, gain of 17q, and *MYCN* proto-oncogene amplification [20, 21]. MYCN status, tumor ploidy, and 11q23 allele status have been included in the International Neuroblastoma Risk Group (INRG) classification system [22]. Recent studies showed that the karyotype changes observed during propagation encompass genomic regions that are frequently altered in human cancer, providing the cancerous cells with a survival or growth advantage [23]. The frequent relapses that are seen in aggressive NB, with decreasing time intervals between relapses, highlight the genetic rearrangements that could drive ongoing acquisition of chemo/radio-resistance and pro-oncogenic adaptations [24, 25]. Identifying the crucial genetic alterations or rearrangements that switch favorable NB to aggressive high-risk NB could lead to the development of an efficient and improved targeted therapeutic strategy and better patient outcomes.

This study used spontaneous and reproduced mouse models of aggressive human NB to document acquired genetic alterations in the NB cells, and further identified the gene manipulations orchestrated as a cause effect. We established clones of distinct populations with aggressive physiognomy (MSDACs), using tumors derived from multiple metastatic sites of various animals. These clones were examined for genetic rearrangements, cancer stem cell (CSC) status, and ability to prompt aggressive disease with systemic metastasis. Clinical outcome association studies in cohorts of neuroblastoma patients showed a strong association of these acquired genetic rearrangements with poor overall and relapse-free survival. For the first time, this study demonstrated the ongoing acquisition of genetic rearrangements and the subsequent switch from favorable NB to high-risk disease, identifying an association between genetic rearrangement, the switch to high-risk disease, and poor clinical outcomes.

## Methods

### Cell culture

The SH-SY5Y human neuroblastoma cell line was obtained from ATCC (Manassas, VA) and was cultured and maintained as described earlier [26]. For passaging and for all experiments, the cells were detached using 0.25 % trypsin /1 % EDTA, resuspended in complete medium, counted (Countess, Invitrogen), and incubated in a 95 % air/5 % CO_2_ humidified incubator.

### Development of neuroblastoma xenografts and mouse model of high-risk metastatic disease

All animal experiments conformed to American Physiological Society standards for animal care and were carried out in accordance with guidelines laid down by the National Research Council. All protocols were approved by the University of Oklahoma Health Sciences Center - Institutional Animal Care and Use Committee. However, Human data used were obtained from public database (http://r2.amc.nl) to demonstrate the significance of altered genes in high-risk disease and their relevance to clinical outcomes. Neuroblastoma xenograft and/or aggressive metastatic disease development was performed as described earlier [27]. Tumor growth, regression, and dissemination to distant sites were investigated by tumor volume measurements and non-invasive fluorescent imaging as described earlier [27]. Animals were euthanized by CO_2_ asphyxiation. The tumors from metastatic sites and non-metastatic xenografts were harvested and prepared as single-cell suspensions as described earlier [27]. To reproduce high-risk aggressive disease, animals were injected with isolated and well-characterized clones of aggressive cells derived from individual metastatic sites, and observed for development of metastatic tumors. Parallel experiments were performed with parental SH-SY5Y cells as describe earlier [27].

### Tumorosphere formation capacity

We plated a total of 10^3^ parental SH-SY5Y cells and MSDACs maintained under *ex vivo* controlled conditions on 100 mm culture plates in serum-free stem cell medium (DMEM:F12 with 1 % N2 Supplement, 2 % B27 Supplement, 20 ng/ml hPDGF, 100 ng/ml EGF, and 1 % antibiotic-antimycotic). Cells were maintained at 37 °C, 5 % CO_2_ for 72 h. We assessed formation of well-organized tumorospheres using phase contrast light microscopy. In parallel, we examined 1000 cells plated in 96-well culture plates with high-content real-time fluorescent time-lapsed video imaging as described earlier [28].

### Routine cytogenetics (G-banding analysis) and array CGH

All cell preparations for cytogenetic analysis, karyotyping, and array CGH were performed in the Cytogenetic Molecular division of the University of Oklahoma Health Sciences Center Clinical Genetics Core. We harvested parental SH-SY5Y cells and MSDACs according to our laboratory standard protocols. Chromosomes were treated and stained by trypsin-Giemsa banding (GTG-banding). A total of 50 cells were analyzed and karyotyped (in a blinded fashion) from each clone of the cell line. For array CGH, genomic DNA was extracted from the parental SH-SY5Y, and aggressive MSDACs by the phenol-chloroform method with slight modifications, as described previously [29]. A total of 1.5 μg of Cyanine 5-dUTP-labeled test DNA and an equal amount of Cyanine 3- dUTP-labeled reference DNA were mixed using a NimbleGen Dual-Color DNA Labeling Kit, and then hybridized to a high capacity NimbleGen CGH array (3 × 1.4 M features, Roche NimbleGen Inc., Madison, WI) according to NimbleGen’s CGH protocols. The arrays were scanned at 532 nm and 635 nm using a NimbleGen MS200 Microarray Scanner. Nexus Copy Number™ software version 7.0 (BioDiscovery Inc., Hawthorne, CA) was used to visualize, detect, and analyze array CGH differences.

### QPCR

We used real-time QPCR to analyze the transcriptional alterations of *ADAM5, A4GALT, APOBEC3B, CD1C, EP300, FOXP2, SLC25A17, L3MBTL2, MAL2, NBPF20, POLDIP3, RALYL*, and *SERHL* (corresponding genes for the observed copy number variation) in SH-SY5Y and in clones of MSDACs grown *ex vivo* as described earlier [26, 30]. We used β*-actin* as a positive control. A negative control without template RNA was also included. Each experiment was carried out in triplicate. The ^ΔΔ^*C*t values were calculated by normalizing the gene expression levels to β*-actin*. The relative expression level was expressed as a fold change over parental SH-SY5Y cells. Group-wise comparisons were performed with two-way ANOVA with Tukey’s post-hoc correction (Prism Version 4.03, GraphPad Software Inc., La Jolla, CA).

#### Immunoblotting

Total protein extraction and immunoblotting were performed as described in our earlier studies [20, 24]. For this study, the protein transferred membranes were incubated with either Rabbit polyclonal RALYL or Goat monoclonal FoxP2 (Abgent, San Diego, CA) and were developed with the appropriate anti-goat/anti-rabbit (BioRad Laboratories, Hercules, CA) secondary antibody.

#### Tissue microarray and, quantitative immunohistochemistry

All tissue microarrays (TMA) IHC staining were performed in the Stephenson Cancer Center Cancer Tissue Pathology Core. To better characterize the correlation between acquired alterations of RALYL to the neuroblastoma progression in clinical subjects, we used a commercially available human neuroblastoma tissue array (Cat. No. MC-602, US Biomax, Inc., Rockville, MD). The 5 μm thick human TMA is equipped with duplicate 1.5 mm cores of neuroblastoma tissues from various sites including the retroperitoneum, mediastinum, abdominal and pelvic cavities, and the adrenal glands of 25 patients. Further, the TMA is armed with clinical variables including sex, age, site/organ, diagnosis, and tumor grading. Pathology diagnosis classification includes: (i) **Grade 1 or well-differentiated -** Cells appear normal and are not growing rapidly; (ii) **Grade 2 or moderately-differentiated** - Cells appear slightly different than normal; (iii) **Grade 3 or poorly differentiated** - Cells appear abnormal and tend to grow and spread more aggressively. H & E stained TMA was reviewed for pathology. For this study, TMA-IHC staining was performed with Rabbit polyclonal RALYL (Abgent). The slides were micro-digitally scanned using an Aperio Scanscope (Aperio Technologies, Inc.,) slide scanner and analyzed using integrated Spectrum software. RALYL nuclear positivity for the cores was then correlated with tumor grades. Group-wise comparisons were performed with GraphPad Prism.

#### Functional characterization of genetic alterations and association to clinical outcomes

We used Ingenuity Pathway Analysis software (Ingenuity Systems, Inc.) to examine the intermolecular interactions and the role that the genes with altered copy numbers and expression played in cancer progression. In this way, we were able to characterize the genetic rearrangements observed to accompany the functional biological response, defined here as tumor progression. We also used the R2: microarray analysis and visualization platform (http://r2.amc.nl) created by Dr. Jan Koster at the Academic Medical Center (AMC), Amsterdam, to examine the association of the observed genetic alterations with overall and relapse-free survival. This web-based application correlates a select gene expression profile with clinical outcomes for samples from various cohorts of patients.

## Results

### Human neuroblastoma (SH-SY5Y) cells with mixed neuroblast-like and epithelial-like cells develop spontaneous high-risk aggressive disease *in vivo*

The subcutaneous administration of human SH-SY5Y cells resulted in the development of ~200 mm^3^ xenografts in ~70 % of the animals within 30 days, as described previously [26, 31], while the other 30 % of the mice were presented with multiple clinically-mimicking aggressive metastatic tumors in the mediastinum and retroperitoneal, pelvic, abdominal, and chest cavities as shown previously (27).

**Fig. 1:**
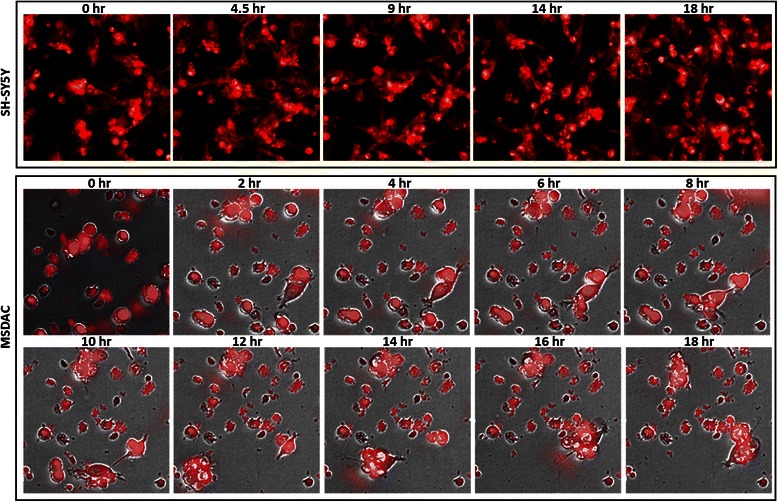
Tumorosphere formation capacity of MSDAC. Representative time-lapse photomicrographs of high-content imaging of parental SH-SY5Y and aggressive MSDACs. Cells were stained with DiI and imaged in real-time every 20 min for 18 h with Operetta. Parental cells (upper panel) showed monolayer spreading, MSDACs (lower panel) showed aggregation and tumorosphere formation

### Aggressive CSC-like MSDACs prompt tumorigenicity and reproduce high-risk disease

To better characterize the established high-risk aggressive disease model and to underscore the enrichment of select clones from the parental line or ongoing acquisition of genetic rearrangements, MSDAC clones were discretely characterized by karyotyping, whole genome array CGH analysis, and tumorosphere-forming capacity. MSDACs are relatively small and spherical with thin neurites. More importantly, every investigated clone of MSDACs exhibited intrinsic CSC characteristics per the ability to readily grow *ex vivo* in serum-free medium and form large organized tumorospheres (28). This process is presumed to simulate the events of tissue regeneration and maintenance from cells that survive suspension conditions. In this process, an initial phase of symmetric expansion of the seeding stem cells precedes a phase of asymmetric division, which gives rise to the differentiated progeny that comprise the sphere bulk. Real-time high-content observation of MSDACs under controlled conditions showed an aggressive aggregation and tumorosphere formation within 18 h (Fig. 1; Additional file 1: video 1 and Additional file 2: video 2). Though parental SH-SY5Y cells and cells derived from non-metastatic xenografts (Fig. 1) survived in serum-free medium, they exhibited monolayer cell spreading without tumorosphere formation. *In vivo*, subcutaneous administration of MSDACs produced relatively large (>500 mm^3^) xenografts as reported earlier [27]. The mice that received MSDACs presented with multiple metastatic tumors in the retroperitoneal, pelvic, abdominal, and chest cavities, demonstrating the reproducibility of the high-risk aggressive disease. Conversely, the mice that received parental cells did not exhibit any distant metastasis, and hence served as the non-metastatic xenograft controls.

**Fig. 2:**
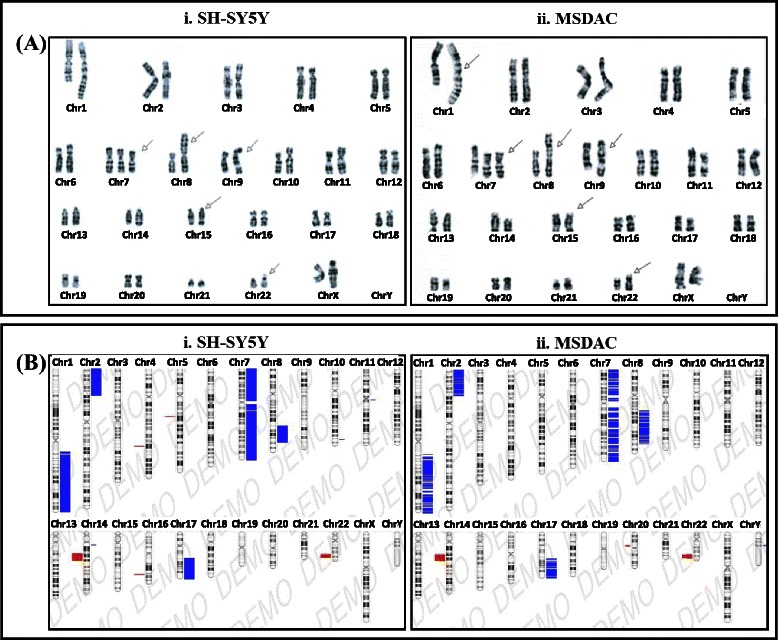
Karyotyping in parental SH-SY5Y and MSDACs. Representative microphotographs showing karyotyping patterns in parental SH-SY5Y and MSDACs by (**a**) G-banding analysis and (**b**) array CGH analysis. G-banding identical *47,XX, add(1)(q32), +del(7)?(q32), add(8)(p23), add(9)(q34), add(15)(q24), add(22)(q13)* [20] karyotyping in SH-SY5Y and MSDACs

### G-banding certified that MSDACs from metastatic mouse tumors are derived from human SH-SY5Y cells

Cancer cells are typically characterized by intricate karyotypes, including both structural and numerical changes. To determine and illustrate that the aggressive tumors developing in multiple metastatic sites were derived from the parental human SH-SY5Y cells, we karyotyped MSDACs, with and without characterized CD133^+^, and compared these with the parental cells. All karyotyping was performed in double blinded fashion. We investigated at least 20 cells per clone. SH-SY5Y cells exhibited the *47,XX, add(1)(q32), +del(7)?(q32), add(8)(p23), add(9)(q34), add(15)(q24), add(22)(q13)* [20] karyotype, and served as the positive controls (Fig. 2*ai*). All investigated clones of MSDACs exhibited an exact match of the parental SH-SY5Y cells. We observed a unique marker composed of a chromosome 1 with a complex insertion of an additional copy of a 1q segment into the long arm, resulting in trisomy of 1q. Karyotyping also revealed six novel non-random chromosomal rearrangements on 1q32, 8p23, 9q34, 15q24, 22q13 (additions), and 7q32 (deletion; Fig. 2*aii*). Consistently, array CGH analysis corroborated the karyotyping in the clones of parental cells and MSDACs (Fig. 2b) and demonstrated that the developed aggressive metastatic tumors in mice are indeed derived and disseminated from the parental SH-SY5Y cells.

**Fig. 3:**
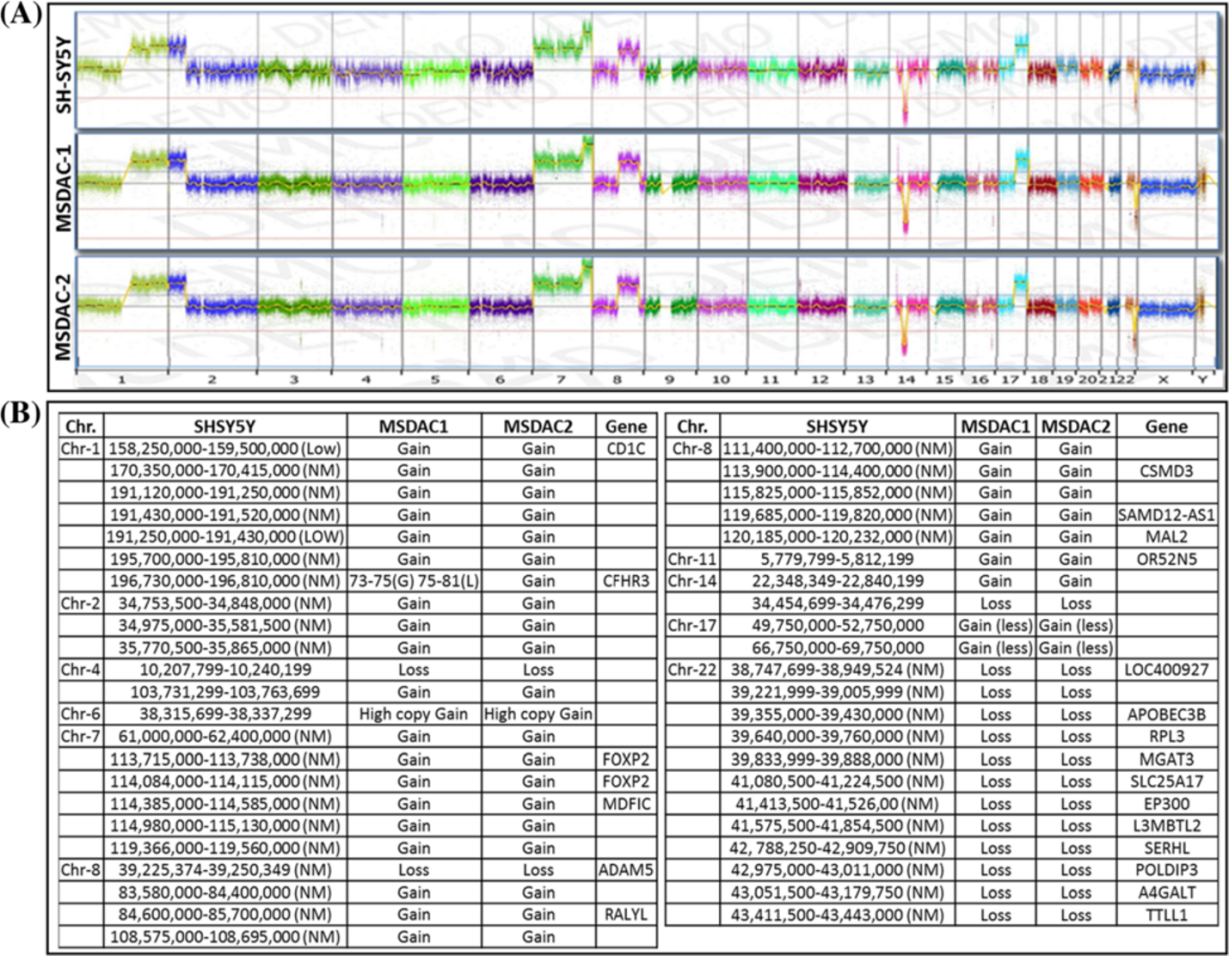
Genome wide copy number variations in parental SH-SY5Y and MSDACs. **a** Array CGH analysis showing digitized copy number variations (CNVs) across the genome plotted for SH-SY5Y cells and MSDACs. **b** Table showing common copy number gain and/or loss across the clones of MSDACs. Chromosome numbers, regions, and magnitude of CNV variation and corresponding genes are shown

### Acquired genetic rearrangements in neuroblastoma cells drive aggressive disease

**Fig. 4:**
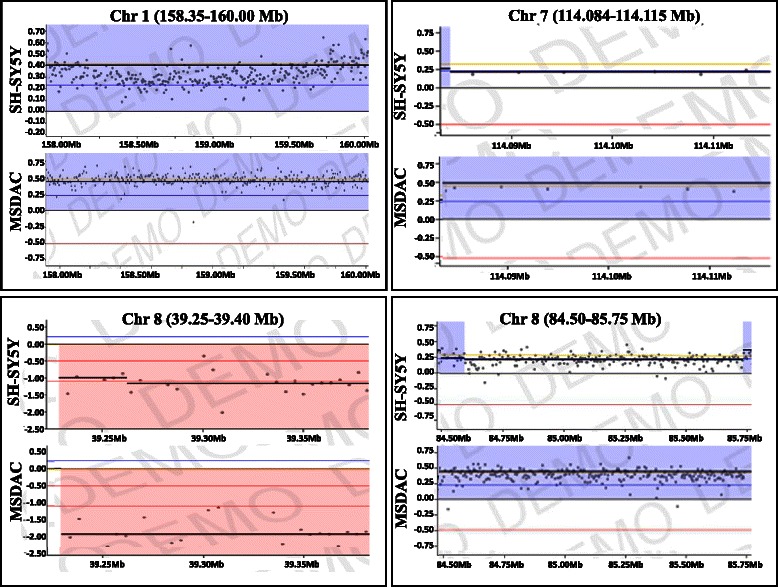
Copy number variations in parental SH-SY5Y and MSDACs. Representative copy number variation charts showing gain in Chr.1, 158.35–160.00 MB; Chr.7, 114.084–114.115 MB; Chr.8, 39.25–39.40 MB, and in Chr.8, 84.50–85.75 MB, corresponding to the coding regions of *CD1C, FOXP2, ADAM5*, and *RALYL,* respectively, in MSDACs compared with SH-SY5Y cells

**Fig. 5:**
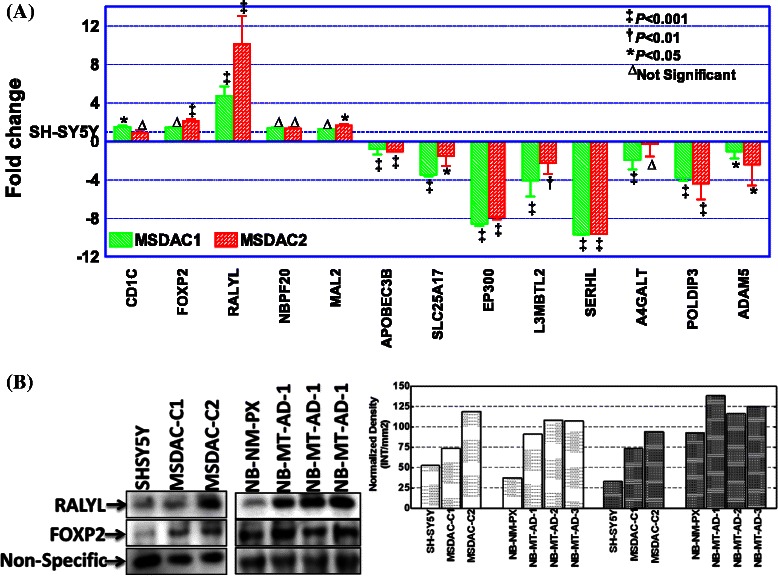
Transcriptional and translational validation array CGH outcomes. **a** Histograms of QPCR analysis showing transcriptional amplification of *CD1C, FOXP2, RALYL, NBPF20,* and *MAL2,* and suppression of *APOBEC3B, SLC25A17, EP300, L3MBTL2, SERHL, A4GALT, POLDIP3,* and *ADAM5* in clones of MSDACs compared with SH-SY5Y cells. **b** Representative immunoblots showing the expression level of RALYL and FOXP2 (both showed gain in Array CGH analysis) in two different clones of metastatic site derived aggressive cells (MSDAC) in comparison with the parental SH-SY5Y cells and in the metastatic tumors derived from three different animals bearing high-risk aggressive neuroblastoma (NB-MT-AD) in comparison with the non-metastatic primary xenograft (NB-NM-PX). Side Panel: Histograms of Quantity one densitometry analysis showing robust increase in RALYL and FOXP2 expression in MSDACs as well as in metastatic tumors *in vivo*

To determine any acquired genetic rearrangements and to underscore their impact on disease progression, we utilized high-throughput whole genome array CGH analysis (Fig. 3a) coupled with quantitative transcriptional expression (QPCR). High resolution array CGH analysis showed unique yet extensive copy-number variations (CNVs), including insertions, deletions, and more complex changes that involve gain (duplication) or loss (deletion) at the same locus in MSDAC clones (Fig. 3a, Fig. 4). However, in order to characterize the association of acquired genetic rearrangements with disease progression, we considered only the common genetic variations across the investigated clones of MSDACs. Forty-five common CNVs were observed with gain in 30 (Chr.1,7; Chr.2, 3; Chr.4, 1; Chr.6, 1; Chr.7, 6; Chr.8, 8; Chr.11,2; Chr.17,2) regions and loss in 15 (Chr.4,1; Chr.8,1; Chr.14,1; Chr.22,12) regions (Fig. 3b, Fig. 4). Interestingly, these CNVs correspond to the gain in the coding regions of *CD1C, CFHR3, FOXP2, MDFIC, ADAM5, RALYL, CSMD3, SAMD12-AS1, MAL2, OR52N5*, *LOC400927, APOBEC3B, RPL3, MGAT3, SLC25A17, EP300, L3MBTL2, SERHL, POLDIP3, A4GALT*, and *TTLL1* genes. (Fig. 3b, Fig. 4)*.* Unlike the healthy genome, in which changes in gene expression are carefully controlled through transcription factors, the cancer genome adapts through the duplication of *CD1C, CFHR3, FOXP2, MDFIC, RALYL, CSMD3, SAMD12-AS1, MAL2,* and *OR52N5*, and loss in the coding regions of *ADAM5, LOC400927, APOBEC3B, RPL3, MGAT3, SLC25A17, EP300, L3MBTL2, SERHL, POLDIP3, A4GALT*, and *TTLL1* genes. QPCR analysis revealed a CNV gain with a corresponding increase in transcriptional expression of *CD1C, FOXP2, RALYL*, and *MAL2* in MSDACs, but not in SH-SY5Y cells (Fig. 5a)*.* Likewise*,* we observed a transcriptional repression of *ADAM5, A4GALT, ABPOBEC3B, EP300, L3MBTL2, SERHL, SLC25A17*, and *POLDIP3*, consistent with the CNV loss in MSDACs (Fig. 5a). Moreover, immunoblotting analysis revealed a profound increase in RALYL and FOXP2 translation in aggressive MSDAC clones as opposed to the parental SH-SY5Y cells (Fig. 5b). Like-wise we observed a robust increase in RALYL and FOXP2 expression in metastatic tumors compared to the non-metastatic primary xenograft (Fig. 5b). Quantity one densitometry analysis revealed consistent increase in RALYL and FOXP2 expression both in *ex vivo* and *in vivo* settings (Fig. 5b side panel). Together, the definite genetic changes (CNV loss/gain) in the coding regions of specific genes and their subsequent transcriptional/translational modulations across MSDACs highlight the acquired genetic rearrangements in neuroblastoma progression.

**Fig. 6:**
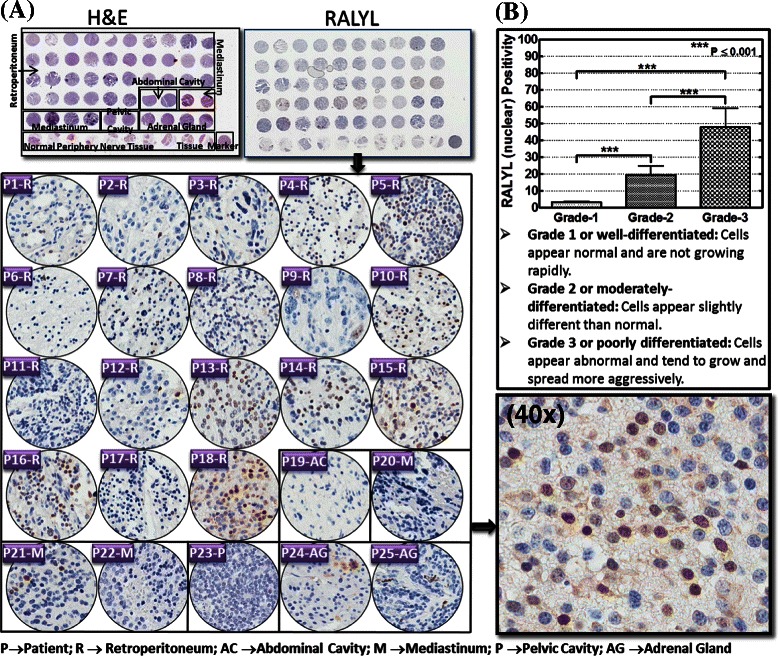
Tumor grade associated expression of RALYL in human neuroblastoma. **a** Thumbnail and constructed images (20×) of human neuroblastoma tissue microarray coupled with automated IHC showing RALYL expression levels in human neuroblastoma samples (n = 25). **b** Aperio image analysis of the TMA and RALYL positivity quantification and subsequent correlation of RALYL expression with neuroblastoma tumor grading

### Acquired alterations associates with poor prognosis

To further substantiate our findings in clinical settings, we examined whether gain/loss in the expression of such candidates correlates with high-risk neuroblastoma utilizing a commercially available human neuroblastoma TMA. The tissues are derived from sites including the retroperitoneal, abdominal, and pelvic cavities, the mediastinum, and the adrenal glands. RALYL-IHC analysis revealed a significant distinction in RALYL staining between patient samples (Fig. 6a). RALYL IHC revealed nuclear positivity with variable levels the human neuroblastoma tissue cores analyzed. Positive RALYL staining appeared in brown and was selectively localized in the nucleus (see 40× panel, Fig. 6a). Correlating the RALYL positivity to the tumor grade clearly identified the directly proportional tumor-grade → RALYL expression association (Fig. 6b). RALYL positivity was relatively low in Grade 1, while its expression increased per increased tumor invasive potential, with maximal gain in highly invasive tumors (Fig. 6b).

### Acquired genetic alterations are associated with tumor progression and poor clinical outcomes

To underscore the importance of the observed genetic rearrangements in aggressive disease, we first clarified their biological functions, network and communal molecular orchestrations, and their documented role in any tumor progression systems. IPA “pathway interaction analysis” revealed a complex yet well-organized signal transduction network of *MAL2, A4GALT, POLDIP3, RPL3, EP300, CD1C, CFHR3, APOBEC3B, RALYL, NBPF20, FOXP2, MDFIC, TTL1*, and *MGAT3* (Additional file 3: Figure S1). Evidently, genes with genetic rearrangements in coding regions play concomitant roles in multiple tumor systems, such as chronic myeloid leukemia, melanoma, small cell carcinoma, lung carcinoma, mammary tumor, prostate cancer, pancreatic cancer, colon adenocarcinoma, squamous cell carcinoma, and non-small cell lung adenocarcinoma. Moreover, “IPA-Core-Analysis” revealed that this small subset of tightly inter-regulated molecular targets showed influential participation in many canonical signaling pathways and demonstrated defined roles in multifarious biological functions. IPA-data mining considering only relationships where confidence = experimentally observed, these molecules exhibited their role in at least 67 different canonical pathways exerting >150 biological functions. Interestingly, in the light of tumor progression and dissemination, we observed a significant association of these molecules in key pathways of cancer progression viz., ATM Signaling, cAMP-mediated signaling, Cell Cycle:Checkpoint Regulation, CREB Signaling in Neurons, Dendritic Cell Maturation, EIF2 Signaling, ERK/MAPK Signaling, ERK5 Signaling, Estrogen Receptor Signaling, FGF Signaling, FLT3 Signaling in Progenitor Cells, G-Protein Coupled Receptor Signaling, Granzyme A Signaling, HIF1a Signaling, ILK Signaling, Neurotrophin/TRK Signaling, NFkB Signaling, p38 MAPK Signaling, p53 Signaling, Phospholipase C Signaling, PPAR Signaling, PPARa/RXRa Activation, Protein Kinase A Signaling, RAR Activation, Pyrimidine Deoxyribonucleotides, TGF-b Signaling, VDR/RXR Activation, Wnt/Ca + pathway, Wnt/b-catenin Signaling *etc.*, (Additional file 4: Figure S2A). In addition to their role in molecular signaling events, these molecules also exercise their defined (*P* < 0.05) roles in cancer progression related bio-functions including Cancer Cell Morphology, Progression of tumor, Cell Cycle-replicative senescence, Cellular Assembly DNA Replication, Cell Cycle arrest, Cell Death and Survival, Cellular Function and Maintenance, Post-Translational Modification, Cell-To-Cell Signaling, Cellular Assembly/Organization, Cellular Growth and Proliferation, Cellular Movement, Cellular Response to Therapeutics *etc.*, (Additional file 4: Figure S2B). To that note, all-encompassing overview of these molecules including information on their symbol, name, subcellular location, protein functions, binding, regulating, regulated by, targeted by miRNA, role in cell, molecular function, biological process, cellular component, disease, role in tumor progression and metastasis *etc.*, are provided in Additional file 5: Table S1.

To demonstrate the relevance of these genetic rearrangements to high-risk neuroblastoma and poor clinical outcomes, we examined the correlation of individual gene expression with overall (OS) and relapse-free survival in patients with neuroblastoma. We utilized a web-based microarray analysis and visualization platform (http://r2.amc.nl) that correlates a select gene expression profile with clinical outcomes for samples from multiple cohorts of patients with neuroblastoma. Kaplan-Meier plots showed a significant association between increased expression of *CFHR3, MDFIC, CSMD3, FOXP2*, or *RALYL* (genes with gains in coding regions) and poor OS in patients with neuroblastoma (Additional file 6: Figure S3A). This inverse association of *CFHR3-, MDFIC-, CSMD3-, FOXP2-*, or *RALYL-*gain also reflects poor relapse-free survival in these patients (Additional file 6: Figure S3A). Interestingly, *SLC25A17, POLDIP3, SERHL, LOC400927, MGAT3*, or *TTLL1* (genes with CNV-loss in coding regions) demonstrated a definite association with their loss and poor OS (Additional file 6: Figure S3B). The loss in any of these genes individually results in poor relapse-free survival in children with neuroblastoma (Additional file 6: Figure S3B). Clinical outcome association analysis also revealed a strong correlation between the expressional variations of both groups of genes listed above and stage progression, favorable → unfavorable disease and alive → died-of-disease (data not shown). It is pertinent to mention that gains in *CD1C, NBPF20,* and *MAL2*, and losses in *ADAM5, RPL3, L3MBTL2, A4GALT, EP300*, and *APOBEC3B* were not associated with poor clinical outcomes (Additional file 7: Figure S4). Together, these data demonstrate the direct, definite influence of genetic rearrangements in aggressive disease on poor clinical outcomes in children with neuroblastoma.

## Discussion

The most devastating aspect of high-risk neuroblastoma is the hematogenous metastasis that produces frequent relapse, evades intense multi-modal therapy, and contributes to death in patients with this disease. Since cancer progression is attributed to the ongoing accumulation of genetic alterations in tumor cells [33], it is critical to describe the genetic rearrangements that prompt and orchestrate the switch from favorable to aggressive high-risk neuroblastoma. For the first time, this study identified the acquired genetic rearrangements in highly malignant populations of neuroblastoma cells that reproduced clinically-mimicking aggressive disease. We found that the acquired genetic rearrangements in these cells correspond to the coding regions of a unique set of genes, and further translate to the altered transcriptional and translational regulation of these genes. Strikingly, we found a strong association of these accumulated genetic rearrangements with poor overall and relapse-free survival.

High-resolution whole genome array CGH analysis identified a distinctive set of genetic rearrangements (gain/loss) that were common across the highly malignant MSDACs. Over the last two decades, identification of numerous oncogenes and tumor suppressors has aided the study of genetic alterations in cancer cells and helped us understand tumor progression and metastasis [34, 35]. Outcomes from studies using large cancer databases illustrated that accumulated genetic alterations may drive phenotypical and biological heterogeneity in tumor cells [3, 36]. Moreover, studies have shown that highly malignant cells often acquire alterations in more genes than do non-metastatic cells; metastatic and non-metastatic cells also express genes differently [37, 38]. In this study, we observed acquired genetic alterations in the coding regions of *CD1C, CFHR3, FOXP2, MDFIC, RALYL, CSMD3, SAMD12-AS1, MAL2,* OR52N5, *ADAM5, LOC400927, APOBEC3B, RPL3, MGAT3, SLC25A17, EP300, L3MBTL2, SERHL, POLDIP3, A4GALT*, and *TTLL1*. Despite the extensive correlation studies that have been previously conducted, to our knowledge this is the first study that identifies the accumulated genetic rearrangements in this setting.

Cytogenetic analysis now extends beyond the simple description of the chromosomal status of a genome, and allows the study of in-depth essential biological questions, including the nature of inherited syndromes, genomic changes involved in tumorigenesis, and three-dimensional organization of the human genome [39]. The results presented here show the robust tumorosphere-forming capacity of MSDACs *ex vivo*. Further, the results provide evidence of aggressive tumor-forming potential with multiple metastases of MSDACs *in vivo*. These outcomes illustrate the clonal enrichment of a select genetically modified, highly malignant sub-population disseminating to distant sites and promoting aggressive disease. To understand the acquired genetic rearrangements in these cells, array CGH coupled with QPCR and immunoblotting are ideal tools. Since cancer stem cells play an instrumental role in cancer relapse and tumor progression [40, 41], exhibition of CSC status in these highly malignant cells further confirms the accumulation of genetic rearrangements and the subsequent drive from favorable to aggressive NB.

Substantiating our findings of acquired genetic rearrangements in the development of aggressive disease, both G-banding and array CGH results confirmed the parental SH-SY5Y cell derivation. Human SH-SY5Y cells are a unique neuroblastoma line composed of N-type and S-type cells [32]. Since the discovery of tumor cell heterogeneity, indicating that a primary tumor often contains sub-populations of metastatic and non-metastatic cancer cells [42], a plethora of evidence corroborating cell heterogeneity to metastatic potential has been found in many tumor systems [43]. In this study, we established clones of MSDACs with high-metastatic potential from a manifold of tumors from metastatic sites of various mice. These cells exhibited CSC physiognomy with ready growth in serum-free conditions and well-organized tumorosphere formation. In addition, the cells reproduced clinically relevant aggressive disease with multiple metastases. More importantly, the results showed differential CNV loss or gain and corresponding gene/protein expression in metastatic MSDACs and non-metastatic cells. Since the differentially expressed genes will induce or suppress tumor progression [33], it is crucial to analyze both gain and loss. For the first time, this study identified a strong clinical outcome association with both CNV gain/increased gene expression and CNV loss/suppressed gene expression. However, the acquired gain in *CFHR3, MDFIC, CSMD3, FOXP2*, or *RALYL* and loss in *SLC25A17, POLDIP3, SERHL, LOC400927, MGAT3*, or *TTLL1* drive poor patient outcomes.

To better understand how cancer cells acquire aggressive metastatic potentials, we must clarify the causative genetic alterations unique to cancer cells with metastatic ability [37, 38]. Researchers have hypothesized that a number of oncogenes or tumor suppressors that are genetically altered in cancer cells undergo ongoing accumulation during tumor progression, and are causative events for multistage carcinogenesis. However, no single oncogene, tumor suppressor, or gene group has been shown to be responsible for the acquisition of invasiveness and metastatic potential in cancer cells. This line of study requires a bidirectional molecular approach, including identification of the genes whose genetic alterations accumulate during cancer progression and identification of genes whose expression is responsible for the acquisition of metastatic potential in cancer cells. We believe the results presented here comprehensively address both questions in the setting of high-risk aggressive neuroblastoma, where (a) genetic rearrangements with array CGH, QPCR and immunoblotting identified the genes whose genetic alterations accumulate during neuroblastoma progression and (b) clinical outcomes (overall survival) association studies as well as clinical tumor grade-correlated RALYL expression identified genes whose expression may be responsible for the acquisition of aggressive disease.

## Conclusions

In conclusion, the results of this study show an accumulation of genetic rearrangements in neuroblastoma cells that drive high-risk aggressive metastatic disease. Specifically, there is CNV gain in the coding regions and conforming expressions of *CD1C, CFHR3, FOXP2, MDFIC, RALYL, CSMD3, SAMD12-AS1, MAL2,* and *OR52N5*, and CNV loss in coding regions and associated regulation of *ADAM5, LOC400927, APOBEC3B, RPL3, MGAT3, SLC25A17, EP300, L3MBTL2, SERHL, POLDIP3, A4GALT*, and *TTLL1.* Highly malignant MSDACs that were derived from metastatic sites exhibited CSC status and exerted robust tumorosphere formations *ex vivo.* These MSDACs also initiated and reproduced high-risk aggressive metastatic disease *in vivo.* Clinical outcome association analysis recognized and identified a strong association with the gain in *CFHR3, FOXP2, MDFIC, RALYL*, or *CSMD3* and loss in *SLC25A17, SERHL, POLDIP3 LOC400927, MGAT3*, or *TTLL1* and poor overall and relapse-free survival. This study described the novel genetic alterations that accumulate during neuroblastoma progression, and defined the role of acquired genetic rearrangements in the clinical outcomes of children with high-risk aggressive metastatic neuroblastoma.

## Additional files

Additional file 1: **Video-1.** Representative reconstructed video from time-lapsed photomicrographs of high-content imaging parental SH-SY5Y cells. Cells were stained with DiI and imaged in real-time for every 20 min for 18 h with Operetta. 
Additional file 2: **Video-2.** Representative reconstructed video from time-lapsed photomicrographs of high-content imaging of aggressive MSDACs. Cells were stained with DiI and imaged in real-time every 20 min for 18 h with Operetta. MSDACs showed aggregation and tumorosphere formation.
Additional file 3: Figure S1. Inter-regulation and network of array CGH identified molecules: Ingenuity pathway analysis showing the interplay of the gene that were identified to have corresponding copy number gain or loss with array CGH analysis, including MAL2, A4GALT, POLDIP3, RPL3, EP300, CD1C, CFHR3, APOBEC3B, RALYL, NBPF20, FOXP2, MDFIC, TTL1, and MGAT3.
Additional file 4: Figure S2. IPA core analysis classification of tumor progression/dissemination related canonical pathways and bio function of array CGH identified molecules: **(A)** Histograms of IPA-data mining considering only relationships where confidence = experimentally observed, showing significant association of array CGH identified molecules in key canonical signaling pathways related of cancer progression. **(B)** Histograms of IPA-data mining (only relationships where confidence = experimentally observed) showing roles of array CGH identified molecules in in cancer progression related bio-functions.
Additional file 5: Table S1. Comprehensive encompassing overview of array CGH identified molecules including the information on their symbol, name, subcellular location, protein functions, binding, regulating, regulated by, targeted by miRNA, role in cell, molecular Function, biological Process, cellular component, disease and, role in tumor progression and Metastasis.
Additional file 6: Figure S3. Correlation of ‘gain’ in *CFHR3, FOXP2, MDFIC, RALYL,* or *CSMD3* and ‘loss’ in *SLC25A17, SERHL, POLDIP3 LOC400927, MGAT3*, or *TTLL1* with clinical outcomes in samples from NB patients: Gene expression-associated clinical outcomes were assessed using the web-based R2: microarray analysis and visualization (http://r2.amc.nl) platform. **(A)** Kaplan-Meier curves computed for a cohort of 88 neuroblastoma patients showing decreased overall and relapse-free survival in patients with high levels of *CFHR3, FOXP2, MDFIC, RALYL*, or *CSMD3*. **(B)** Kaplan-Meier curves computed for a cohort of 88 neuroblastoma patients showing decreased overall and relapse-free survival in patients with low levels of *SLC25A17, SERHL, POLDIP3 LOC400927, MGAT3*, or *TTLL1*.
Additional file 7: Figure S4. Kaplan Meier plots showing clinical outcomes in a cohort of 88 neuroblastoma patients in association with the expression pattern of *CD1C, NBPF20, MAL2* (observed copy number gain in the current study) and *ADAM5, A4GALT, RPL3, L3MBTL2, APOBEC3B* and *EP300* (observed copy number loss in the current study).
