# Supplementary figures and images for: Acquired genetic alterations in tumor cells dictate the development of high-risk neuroblastoma and clinical outcomes
Source: BMC Cancer. 2015 Jul 10;15:514. doi: 10.1186/s12885-015-1463-y (PMC4496850; doi:10.1186/s12885-015-1463-y)

## Slide 1
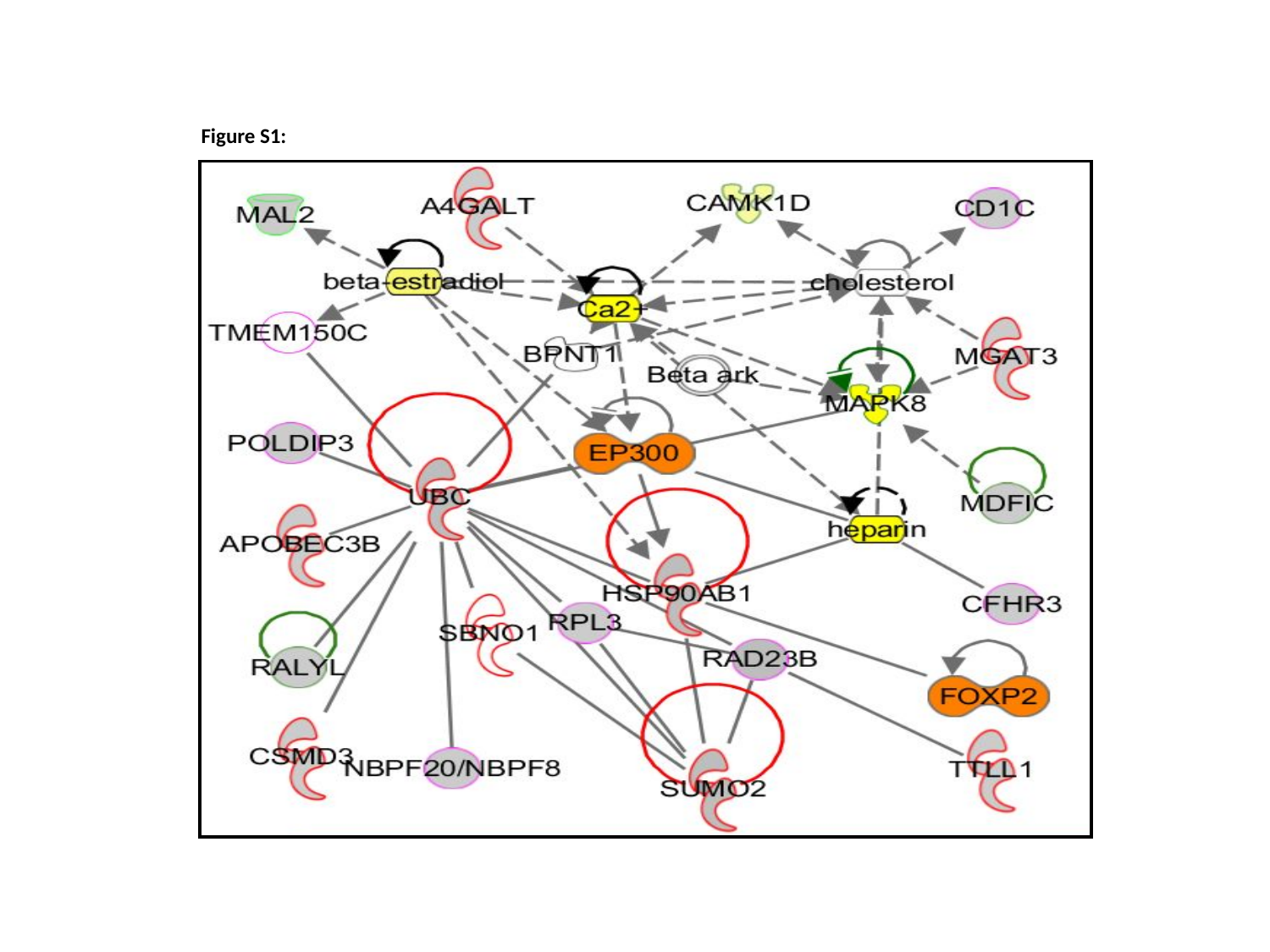

Figure S1:

Supplement: Additional file 3: Figure S1. — Inter-regulation and network of array CGH identified molecules: Ingenuity pathway analysis showing the interplay of the gene that were identified to have corresponding copy number gain or loss with array CGH analysis, including MAL2, A4GALT, POLDIP3, RPL3, EP300, CD1C, CFHR3, APOBEC3B, RALYL, NBPF20, FOXP2, MDFIC, TTL1, and MGAT3. [file 12885_2015_1463_MOESM3_ESM.pptx]

## Slide 1
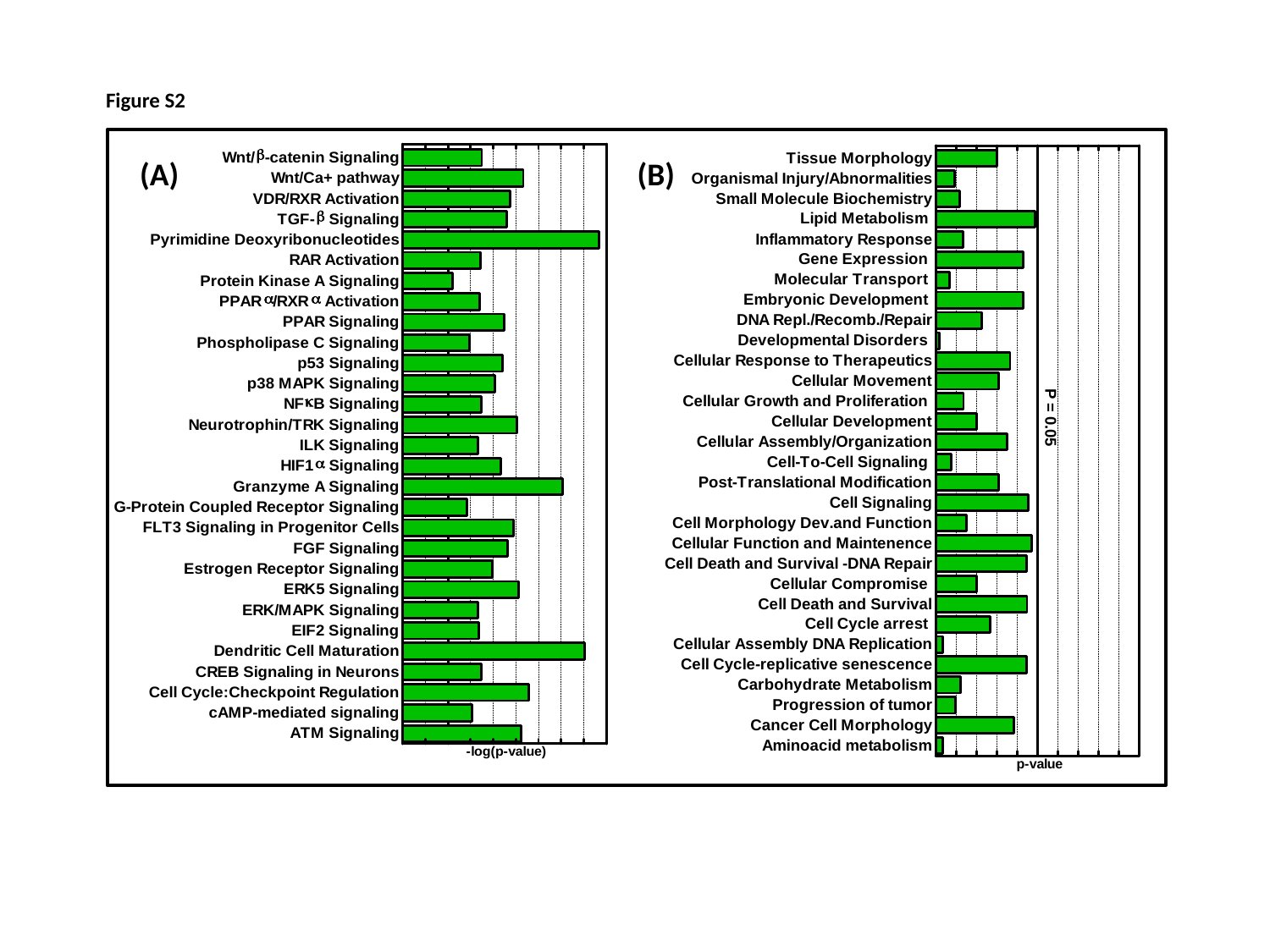

Figure S2
(A)
(B)

Supplement: Additional file 4: Figure S2. — IPA core analysis classification of tumor progression/dissemination related canonical pathways and bio function of array CGH identified molecules: (A) Histograms of IPA-data mining considering only relationships where confidence = experimentally observed, showing significant association of array CGH identified molecules in key canonical signaling pathways related of cancer progression. (B) Histograms of IPA-data mining (only relationships where confidence = experimentally observed) showing roles of array CGH identified molecules in in cancer progression related bio-functions. [file 12885_2015_1463_MOESM4_ESM.pptx]

## Slide 1
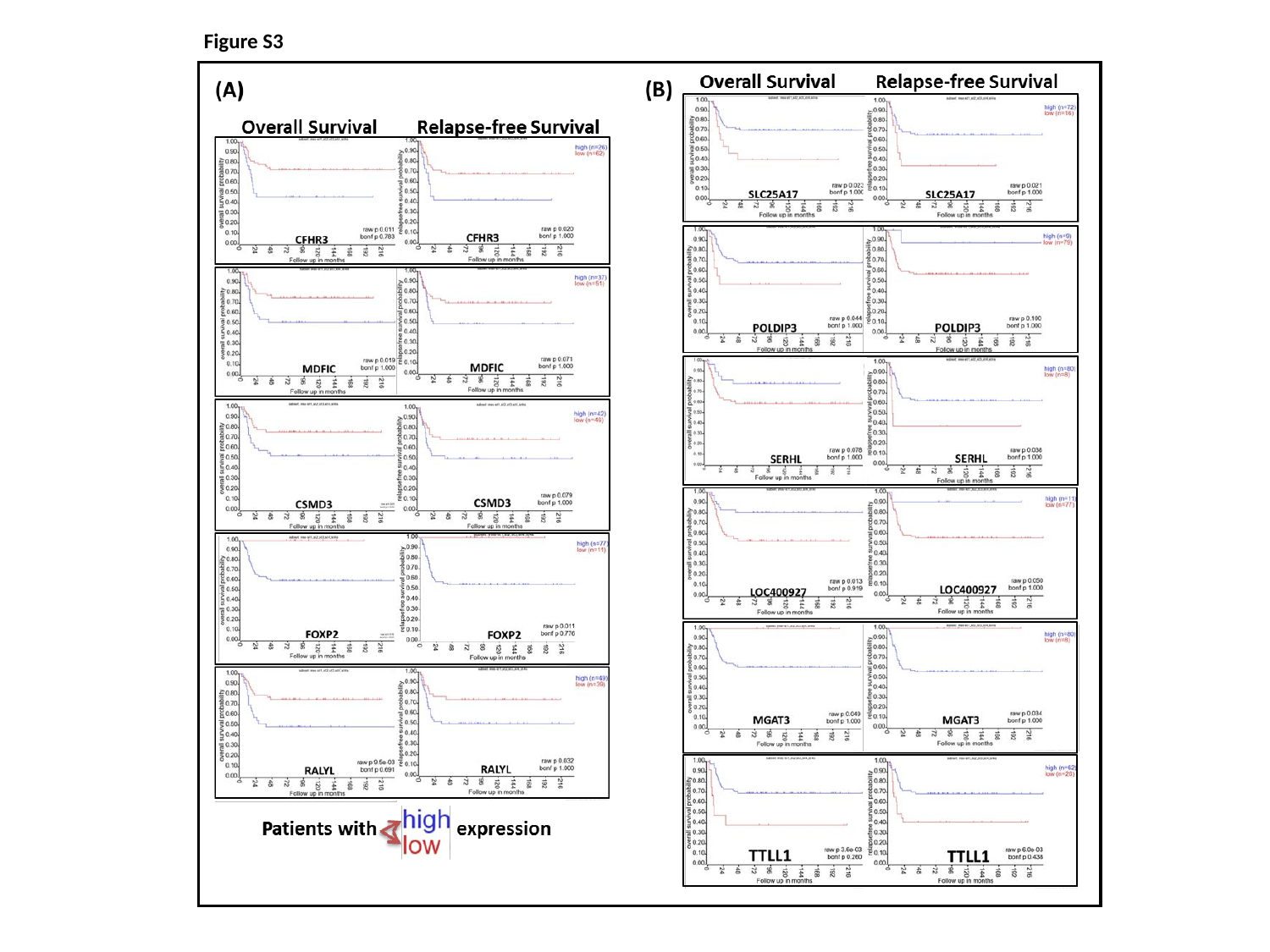

Figure S3

Supplement: Additional file 6: Figure S3. — Correlation of ‘gain’ in CFHR3, FOXP2, MDFIC, RALYL, or CSMD3 and ‘loss’ in SLC25A17, SERHL, POLDIP3 LOC400927, MGAT3, or TTLL1 with clinical outcomes in samples from NB patients: Gene expression-associated clinical outcomes were assessed using the web-based R2: microarray analysis and visualization (http://r2.amc.nl) platform. (A) Kaplan-Meier curves computed for a cohort of 88 neuroblastoma patients showing decreased overall and relapse-free survival in patients with high levels of CFHR3, FOXP2, MDFIC, RALYL, or CSMD3. (B) Kaplan-Meier curves computed for a cohort of 88 neuroblastoma patients showing decreased overall and relapse-free survival in patients with low levels of SLC25A17, SERHL, POLDIP3 LOC400927, MGAT3, or TTLL1. [file 12885_2015_1463_MOESM6_ESM.pptx]

## Slide 1
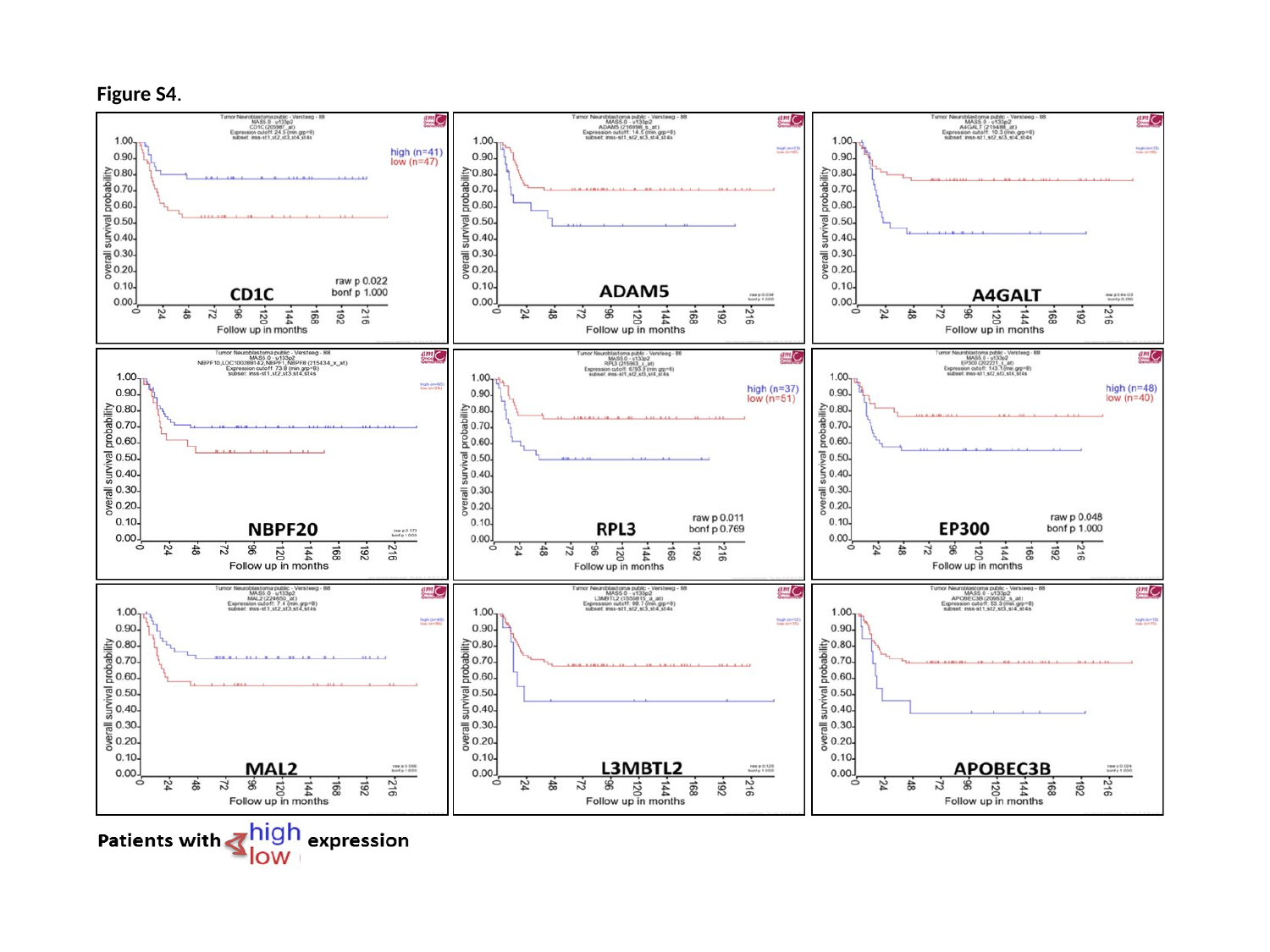

Figure S4.

Supplement: Additional file 7: Figure S4. — Kaplan Meier plots showing clinical outcomes in a cohort of 88 neuroblastoma patients in association with the expression pattern of CD1C, NBPF20, MAL2 (observed copy number gain in the current study) and ADAM5, A4GALT, RPL3, L3MBTL2, APOBEC3B and EP300 (observed copy number loss in the current study). [file 12885_2015_1463_MOESM7_ESM.pptx]
